# The Medical Department of Niagara University

**Published:** 1896-07

**Authors:** 


### THE MEDICAL DEPARTMENT OF NIAGARA UNIVERSITY.

HEREAFTER, the course of study in the Niagara Medical College will be extended to four years, including four courses of lectures.

Several changes have been made in the faculty, among which may be mentioned the following : Dr. Rollin L. Banta has been made associate professor of obstetrics ; Dr. Carlton C. Frederick, associate professor of gynecology and clinical gynecology ; Dr. Harry A. Wood, professor of materia medica and therapeutics and insanity ; Dr. Eugene A. Smith, professor of principles of surgery, in addition to his former position ; Dr. Edward M. Dooley, associate professor of anatomy ; Dr. Walter D. Greene, professor of genito-urinary diseases ; Dr. Louis A. Weigel, of Rochester, lecturer on orthopedy ; Dr. David L. Redmond, lecturer on hygiene ; Dr. Alfred E. Diehl, lecturer on dermatology ; Dr. Sydney A. Dunham, adjunct professor of physiology ; Dr. Lawrence G. Hanley, lecturer on obstetrics ; Dr. John H. Daniels, lecturer on materia medica ; Dr. Fred. S. Hoffman, instructor in anatomy ; Dr. Wm. G. Taylor, instructor in obstetrics. Some other additions will yet be made and announced later.

The faculty has added a new office to its staff—namely, registrar, to which Dr. Hubbell has been elected. Dr. Harry A. Wood becomes secretary of the faculty.

Out of the twelve who graduated from this school at its last commencement, six have received hospital appointments. Drs. Burke, Walton and Mahoney are at the Sisters of Charity Hospital, Dr. Carr is at the Emergency, Dr. Smith at the Erie County, and Dr. Hughes at the State Hospital.

